# The Complexity of Hearing Aid Fitting: Children with Congenital Hearing Loss and Middle Ear Dysfunction

**DOI:** 10.3390/children10101630

**Published:** 2023-09-30

**Authors:** Ronit Priner, Devora Brand

**Affiliations:** 1 Department of Communication Disorders, Hadassah Academic College, Jerusalem 9101001, Israel; ronitpr@edu.hac.ac.il; 2 Hearing and Speech Clinic, Hadassah Medical Center, Jerusalem 9574425, Israel

**Keywords:** congenital hearing loss, pediatric hearing aid fitting, middle ear effusion

## Abstract

Background: The early diagnosis of hearing loss (HL) and hearing rehabilitation facilitate language and communication development. Some children exhibit mixed HL due to middle ear effusion (MEE) or acute otitis media (AOM). Mixed HL can affect HL evaluation and hearing aid (HA) fitting. The present study retrospectively evaluated the prevalence of MEE/AOM among children with congenital sensorineural HL (SNHL) who were fitted with HAs and its effect on the HA fitting. Methods: Thirty-six HA fittings carried out between 2017 and 2020 at one rehabilitation center were examined. Medical and audiological information was retrieved for children between 6 and 32 months old. The number of appointments and HA fitting times were recorded. Results: Twenty-eight children were included in the study. Eighteen children, in addition to SNHL, had a conductive component resulting from MEE/AOM. The children with these pathologies required significantly more HA fitting sessions and hearing tests, fewer real ear to coupler difference (RECD) measurements and longer HA fitting periods. Conclusion: The findings indicate that a large number of children fitted with HAs have an additional conductive component that makes the fitting process longer. Since early rehabilitation is necessary for language development, otolaryngologists should be aware of the adverse effects of MEE/AOE on the HA fitting process. It is important to inform parents that when there is a conductive component, the HA fitting process may take longer and that treatment by an otolaryngologist is vital. This study stresses the importance of multidisciplinary cooperation for optimal HA fitting.

## 1. Introduction

Congenital hearing loss (HL) has implications for the hearing development of the child, as well as their language, academic and social development [1,2]. The prevalence of congenital HL is 1–3 for every thousand births [3,4]. Newborn hearing screening is mandated in many countries, as recommended by the JCHI (Joint Committee on Infant Hearing) for diagnosing permanent congenital HL [5].

The decision regarding hearing aid (HA) candidacy and amplification is based on the child’s hearing thresholds, which are typically established through such electrophysiological tests as the auditory brainstem response (ABR) [6]. The presence of a conductive component may compromise the reliability of this test in assessing hearing thresholds [7]. A conductive component can occur for various reasons, including middle ear effusion (MEE), with or without acute otitis media (AOM). 

The prevalence of an HL derived from MEE, with or without AOM, varies by age [8,9]. MEE in children necessitates further hearing evaluations and may also prolong the identification of a sensorineural HL (SNHL). Middle ear (ME) pathology combined with congenital HL can pose a challenge for audiologists in their hearing evaluation and rehabilitation procedures, thus causing a delay in optimal hearing rehabilitation for the child with such a need [10].

### 1.1. Early Detection of HL and HA Fitting

Extensive information has accumulated regarding the effects of HL on language development, reading, auditory memory, life quality measures and occupational skills [2,11,12]. The importance of early hearing habilitation has been well established as well [2,13]. JCHI recommends for all infants to have their hearing screened before the age of one month. Infants diagnosed with permanent HL are referred to audiological habilitation before the age of three months [14]. A reliable hearing test is necessary for the accurate fitting of an HA. Since babies and toddlers are not always able to cooperate and provide a full behavioral hearing test, the ABR test is often used to determine the extent and type of the HL [15]. However, this test has its limitations, especially in infants’ measurements and in conductive HL. Research has consistently found a deviation between the thresholds obtained by tonal stimuli ABR and behavioral thresholds, hence the need for correction factors [16,17,18]. Behavioral testing is always preferred, despite the young age, because of the limitations of physiological tests due to the calibration and temporal integration issues [19]. The importance of evaluating the type and severity of the HL is essential for determining the infant’s candidacy for HA fitting and its amplification [20]. Mixed losses can impact on the reliability of the results of the evaluation of HL, as well as the HA fitting amplification so that ME pathologies may challenge the fitting process. 

### 1.2. ME Effect on HA Fitting

The two HA fitting formulas in greatest use for pediatric HA fitting are NAL-NL2 and DSL-v5 [21]. Both of these formulas take into consideration the type of HL. For conductive components, more amplification is prescribed in comparison to SNHL [22]. For this reason, for young children with various degrees of SNHL an additional conductive loss due to MEE/AOM can be critical, since it will impact on the amplification they will need from their HAs. Few studies have looked at conductive pathologies among children with SNHL. Das (1990) [23] found that in a group of children aged two to six, diagnosed with SNHL, 93 (57%) had a conductive component. Abou-Elhamd et al. (2006) [24] evaluated a wider age range, finding a lower percentage, approximately 30%, when examining the presence of MEE for children aged four–twelve years with SNHL. A more recent study of children from newborn to six years with severe to profound HL, who were candidates for cochlear implant surgery, found similar findings to Abou-Elhamd et al. (2006), whereby 26% of the patients had an ME pathology [25]. These findings seem to indicate that younger children have a higher prevalence of ME pathologies. 

There are various treatments available for MEE. Some nonsurgical treatments for pediatric MEE like, auto-inflation and direct eustachian tube inflation which are considered safe and noninvasive [26]. However, these treatments are not always a viable solution for very young children. Recent international guidelines strongly recommended against the use of steroids, antibiotics, and antihistamine drugs. The guidelines indicate clinical monitoring and eventually surgeries, i.e., adenoidectomy, myringotomy or ventilation tube insertion in necessary cases [27].

HA fitting amplification is based on hearing thresholds. When a child acquires a temporary conductive component due to an ME pathology, the fitting process is elongated, since the HA needs to be refitted constantly to the potentially fluctuating hearing thresholds. Additionally, if a child has ventilation tubes (VTs), they can become clogged by secretions or fall out and can leave ear drum perforations that may cause an additional conductive HL [28]. Another example of the impact on the HA fitting process of ME pathologies concerns secretions. It is recommended not to use HAs with discharging ears to allow for the ear to be ventilated and heal, as well as to prevent infections [29]. During this period, the child experiences increased HL due to the conductive component and the absence of regular amplification. At times, this period can take weeks or even months, while, in the meantime, the child may miss out on language exposure, communication and learning opportunities. There does not seem to be a single treatment recommendation to improve the ME function to solve this temporary conductive component of the HL [10].

Despite clinical knowledge of the challenge of the conductive component on the rehabilitation process of children with SNHL, no study has yet addressed the effects on the HA fitting process. The purpose of the current study was to examine the prevalence of MEE and/or AOM among toddlers with permanent SNHL during their HA fitting processes at the rehabilitation center (Micha) and to examine the treatments received for their MEE/AOM. The effects of the MEE/AOM on the HA fitting process were studied as well.

## 2. Methods

### 2.1. Research Procedures

After receiving ethical approval from the board of ethics at Hadassah Academic College and from Micha’s management, a retrospective study was conducted on children who had HAs fitted at the Micha Jerusalem center between 2017 and 2020. Data retrieved from files included demographics, such as age and gender, medical and audiological history, including HL risk factors, HL etiology, visits to the otolaryngologist, otoscopic results, behavioral and physiological hearing tests (ABR and tympanometry), surgical history and VT complications. In addition, we recorded the number of fitting sessions, real ear to coupler difference (RECD) measurements during the fitting, amplification targets and the duration of the HA trial period.

One of the advantages of the current study is that all children were seen and fitted by the same audiologists and with the same HA fitting protocol. In Israel, children up to the age of six are referred for HA fitting in a hospital or in rehabilitation centers. Micha is a chain of pediatric centers with locations throughout Israel. The HA process at Micha in Jerusalem is based on the American Audiology Academy (AAA) and Western Ontario recommendations [30,31]. It must be mentioned that Israel has universal health insurance and every child under eighteen years of age receives government funding for HAs (notice of the Health Administration, 7/2011), and in order to qualify, the audiologist needs to submit a hearing test with and without HAs. HAs are purchased after a successful trial period.

### 2.2. Participants

A total of 36 children with HL underwent HA fitting processes at the Micha rehabilitation center between 2017 and 2020. The exclusion criteria were children that had ME malformation who were referred to an implant center at a hospital for a bone conduction hearing aid consult or candidates for a cochlear implant due to the severity of their SNHL. Eight children were excluded from the study. Three children had a nonconventional pathology (enlarged vestibular aqueduct, or auditory neuropathy), which necessitated deviation from the HA fitting protocol. Five children who were candidates for a cochlear implant and the HA fitting were part of the candidacy process. The medical ear history of the children excluded from the current study was from 16 ears: 1 had no MEE, 5 ears underwent VT surgery, 2 underwent tympanic membrane puncture and 8 had fluid without receiving treatment. The study included a total of 28 children.

### 2.3. Clinical Instruments

Behavioral hearing tests were conducted using an AC-40 Audiometer from Interacoustics with insert phones. An acoustic treated room was used with a digital visual reinforcement. ME function was tested using a Madsen Zodiac Tympanometer. An RECD measurement was conducted with the Audioscan Verifit2.

### 2.4. Statistical Analysis

The mean and standard deviation were calculated for the hearing thresholds, medical and audiological histories, number of appointments and HA fitting time, as well as the amplification data of the patient’s HA. A comparison was calculated using the Mann–Whitney test, *t*-test and χ^2^ test. 

## 3. Results

### 3.1. Treatment for the ME Pathology during the HA Fitting

**Figure 1:**
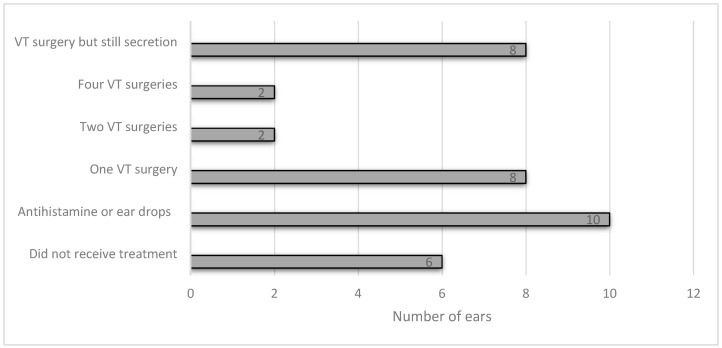
The medical ear history of the study group.

A total of 28 (56 ears) patients with HL who underwent HA fitting processes at the Micha rehabilitation center between 2017 and 2020 met the inclusion criteria. Eighteen children were found to have a medical history of MEE and/or AOM, referred to as the study group, and 10 children had no medical history of ME pathologies, referred to as the control group. The medical ear history of the study group is presented in Figure 1. 

**Figure 2:**
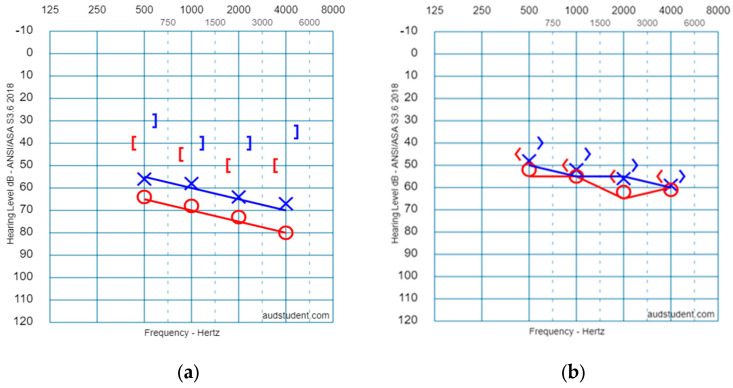
Average thresholds of the behavioral hearing tests for the two groups: (**a**) (right) study group; (**b**) (left) control group.

The age range for the study group was 6–32 months, M, SD = 16.9 ± 8.5 months. The age range in the control group was 6–30 months, M, SD = 19 ± 6.5 in the control group. No significant differences were found between the ages of the two groups (t (27) = 0.73, *p* > 0.05). The study group included ten girls and eight boys, and the control group included five girls and five boys. There was no significant difference in the genders of the two groups, (χ^2^1, 0.05 = 0.038). An average audiogram can be seen in Figure 2 for both groups Figure 2a, for the study group, and Figure 2b for the control group.

The results (average and SD in each group for the AC and BC results) are shown in Table 1.

As shown in Figure 2 and Table 1, the average hearing thresholds in the study group ranged from 62.9 dB to 78.1 dB, with a mixed HL in the right ear. PTA AC = 67.2 dB, PTA BC = 45.4 dB, and it ranged from 55.6 dB to 66 dB mixed HL in the left ear. PTA AC = 59.2 dB, and PTA BC = 40.2 dB. The average hearing thresholds in the control group ranged from 52.5 dB to 62.1 dB SNHL in the right ear. PTA AC = 56.7 dB, PTA BC = 50.1 dB, and it ranged from 47.9 dB to 59.3 dB SNHL in the left ear: PTA AC = 51.9 dB, and PTA BC = 45.2 dB. According to the Mann–Whitney test, there was no significant difference between the groups’ BC thresholds: right ear—500 Hz, *p* = 0.8; 1000 Hz, *p* = 0.96; 2000 Hz, *p* = 0.48; 4000 Hz, *p* = 0.65; left ear—500 Hz, *p* = 0.36; 1000 Hz, *p* = 0.94; 2000 Hz, *p* = 0.48; 4000 Hz, *p* = 0.14. The hearing thresholds for AC were found to be significantly worse in the study group compared to the control group, in the right ear for the frequencies: 500 Hz, *p* = 0.02; 1000 Hz, *p* = 0.02; 4000 Hz, *p* = 0.04. There was no significant difference among the groups’ AC thresholds in the right ear—2000 Hz, *p* = 0.08. In addition, no significant differences were found among the AC thresholds of the left ear between the two groups in all frequencies: 500 Hz, *p* = 0.14; 1000 Hz, *p* = 0.13; 2000 Hz, *p* = 0.16; 4000 Hz, *p* = 0.13.

As can been seen in Table 2, the most prevalent etiology of HL for both groups is genetics. No significant difference in the etiologies of HL was found between the two-group test (χ^2^5, 0.05 = 3.690).

**Figure 3:**
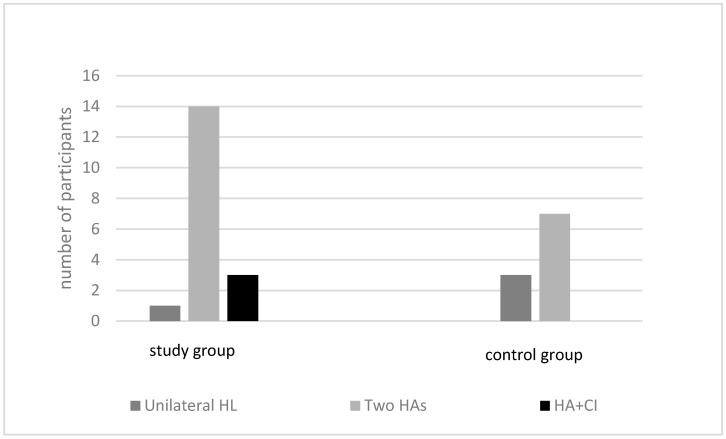
Hearing devices in both groups.

Most children were fitted with two HAs in both groups. Few children had a bimodal fitting of a cochlear implant in one ear and an HA in the other ear, and even fewer children had one HA. There were no significant differences in the hearing devices used in the two groups (χ^2^2, 0.05 = 3.950), as can be seen in Figure 3.

### 3.2. Referrals to an Otolaryngologist

Children were referred to an otolaryngologist if a conductive component was found in the hearing test or an abnormal tympanogram was obtained. The average and standard deviation for such a referral was 4.2 ± 1.8 in the study group and 1.55 ± 1.01 in the control group. The number of referrals to an otolaryngologist during the fitting process was significantly greater in the study group compared to the control group (t (27) = 4.40, *p* < 0.001).

### 3.3. Number of Fitting Sessions

The number of fitting sessions and behavioral hearing tests during the fitting period were reviewed. The average number of fitting sessions was 6.7 ± 2.69 in the study group and 4.0 ± 1.41 in the control group. Children with MEE had significantly more fitting sessions (t (27) = 3.61, *p* < 0.001).

### 3.4. Number of Hearing Tests

The average number of hearing tests was 6.33 ± 2.74 in the study group and 3.7 ± 1.40 in the control group. Children with MEE had significantly more hearing tests (t (27) = 3.50, *p* < 0.001).

### 3.5. RECD Measurement

As part of the fitting process, RECD is measured to provide appropriate amplification for the child. In this study, we checked how many ears could not be tested. There was a significant difference between the groups (χ^2^2, 0.05 = 6.050). In the study group, measurement was not possible for almost half of the ears (14 ears) while, in the control group, this was the case only for two ears.

### 3.6. HA Amplification Gain

**Figure 4:**
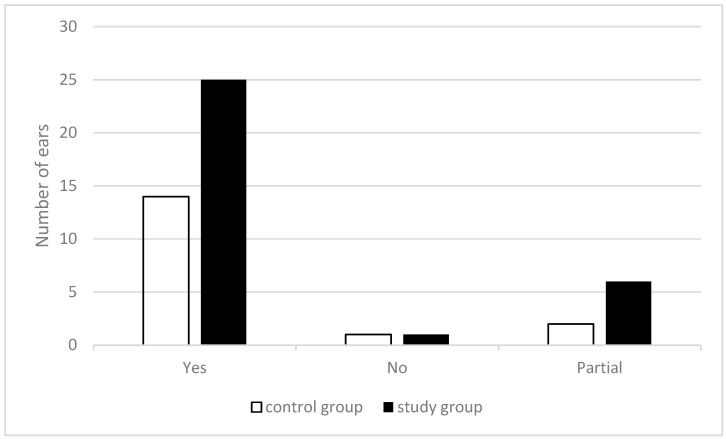
Number of ears that HA gain achieved the targets in both groups.

The HAs were fitted according to formulas based on the child’s hearing thresholds. During the fitting process, we verified that the HA gain achieved the targets with the textbox of the verifit2. In the study group, out of 32 ears, 25 achieved the target, 6 ears achieved it partially, and one ear did not achieve the target at all. In the control group, out of 17 ears, 14 achieved the targets, two ears achieved it partially, and one ear did not achieve the target at all. No significant differences were found for reaching the gain targets in the two groups (χ^2^2, 0.05 = 0.543), see Figure 4.

### 3.7. HA Fitting Process Time

The length of time of the fitting process until the purchase of the device was reviewed. We found a significant difference between the groups regarding the length of the HA fitting process (t (27) = 3.61, *p* < 0.001). Children with MEE had a longer fitting time of 10.29 ± 5.28 months, while for the control group, the fitting process time was 4.6 ± 2.50 months.

## 4. Discussion

The study results show that a large percentage (64%) of the children who were fitted with HAs had an ME pathology in addition to permanent SNHL. The prevalence of a conductive component derived from MEE or AOE in our study was found to be slightly higher than previous studies, where the findings ranged from 26% to 57% [23,24,25]. It is important for pediatric audiologists to be aware of the great incidence of ME pathologies and their effect on diagnosis and rehabilitation.

Regarding treatment, the number of VT surgeries in this study seems to be slightly higher than what was reported in previous studies. According to these studies [23,24,25], 35–42% of patients diagnosed with MEE or AOE underwent VT surgeries. In our study, the percentage was 55%. The higher number of surgeries in our study may be because of the public health care that is available in Israel and perhaps easier accessibility to medical services and care.

Full behavioral hearing tests for all frequencies, BC and AC, were not available for all of the children in this study because of their young age and limited cooperation. However, since there is a preference for behavioral testing over ABR for fitting HAs, this test was prioritized for the fitting process and is presented in this study. In most cases, the children had, at least, a partial behavioral test. Our findings show that no conductive component was found in the control group without ME pathologies in contrast to the study group, where the conductive component ranged from 17.5 to 26.9 dB due to the ME pathologies. Brookhouser et al. 1993 [32] found similar ABG values of 24.5–29.5 dB. We found that although there is no significant difference among the BC thresholds of the two groups, there was a significant difference among the AC thresholds of the two groups for the right ear thresholds. We assume that similar findings were not found for the left ear due to the fewer AC thresholds available for comparison. Generally, behavioral hearing testing starts with the right ear. A temporary conductive HL in addition to permanent loss may inhibit language development and compromise amplification. More gain is needed in the case of a conductive component. However, if the ME resolves, the provided amplification becomes too loud. Therefore, it is crucial to have frequent hearing tests and fine tune the HAs accordingly, and the intervention of the otolaryngologist becomes even more significant.

Therefore, it is not surprising that the number of referrals to an otolaryngologist was found to be significantly higher among children with ME pathologies. This, of course, can add additional stress, responsibility and time demands on parents [33] and, as seen in this study, prolongs the rehabilitation process. It seems from this study’s findings that there is no single optimal treatment for ME fluid or ear infections. Westerberg et al. (2005) [10] claims that the existing literature does not refer to children of these ages with a combination of a permanent SNHL in addition to a temporary HL due to the presence of MEE. They recommend aggressive treatment: prophylactic antibiotics for children with coexisting SNHL with MEE to prevent infections, since such children are also at a greater risk of negative consequences of having a conductive HL. In addition, they recommend choosing the more invasive treatment options, such VT, without delay. It is important to note that this procedure may have frequent and severe complications [26], and there is little evidence of its effectiveness [27]. The guidelines for treating a population with fluids have not changed since 2005—and they may need to be reevaluated. At a conference for experts in otolaryngology specializing in infants, Liming et al. (2016) [34] presented a chart for detecting potential HL. They suggested that when an infant up to the age of three months has persistent fluids for three months, or for four to six weeks if he is older, myringotomy treatment should be provided, or, alternately, insertion of a VT. They asserted that an ABR test is required prior to medical intervention to ensure that the HL is solely conductive and that hearing rehabilitation is not required. In addition, if there is a sensorineural component, the treatment should be conducted more quickly. They indicated surgery as being necessary if there is a conductive HL of at least 20 dB before the age of three years and older with a delayed speech development, which is one of the results of having an HL [35]. The lack of clear clinical guidelines seems to lead to a variety of treatments offered by otolaryngologists for children with fluids.

There are no published guidelines to our knowledge that specifically refer to children with mixed HL. Since there is no single treatment that provides an optimal solution for ME pathologies, we believe it essential to develop guidelines and protocols for the management and treatment of ME pathologies for children with SNHL.

As seen in this study, having ME pathologies adds a significant conductive component that requires additional fitting appointments and gain, compared to children with only SNHL. In addition, it may be that the HA fittings are less precise for children with mixed HLs, since RECD measurements are not always possible—as was found in this study. The presence of fluids or a VT affects the resonance of the external ear canal which, in turn, affects the amplification that reaches the ear drum—which is the reason why RECD measurements are needed for accurate amplification. When measurements are not possible, averages of the ear canal volumes of the child’s age are used. However, using the average measurements at this age is not advised [36], since there are large deviations that can significantly affect amplification. Therefore, a solution for the conductive component is required, not only because of the additional conductive HL but to accurately prescribe amplification.

As mentioned, these complications also lead to a lengthier HA fitting process. If a conductive component is found, parents need to be notified that the fitting process may take longer, with more appointments needed with the otolaryngologist and the audiologist for HA fitting sessions.

This study uncovers the complexity of fitting HAs for children who have a temporary conductive component in addition to permanent SNHL. The aforementioned data are another example of why multidisciplinary cooperation is required for an efficient HA fitting process. Cooperation among the otolaryngologist, rehabilitating speech therapist, fitting audiologist, educational team and parents is essential. The better the communication, the more efficient the HA fitting and rehabilitation processes will be. In addition, as mentioned above, clear criteria are needed for the otolaryngologist treatments for this population, which is different from children with typical hearing and ME pathologies. The limitations of this study are the relatively small number of children that were included, perhaps not a representative sample, since all of the children were from only one center, in one city. In addition, there was a lack of full behavioral hearing tests because of the age and cooperation abilities, as well as the multiple types of etiologies of HL. Further studies on a larger number of children with similar etiologies of HL could be beneficial to further understanding the effect of mixed HL on the HA fitting process. Examining the treatment efficiency of MEE and its conductive component should be examined. The clinical implications of the aforementioned study are the need for clear clinical guidelines for treating MEE/AOM and close and continuous monitoring of the pathology, with post-surgery follow-up.

## 5. Conclusions

Many children with permanent SNHL have additional temporary conductive HL, which results in more visits to the otolaryngologist, more HA fitting sessions and hearing tests, and a longer HA fitting process in general. An updated treatment protocol for otolaryngologists is necessary for these situations. Hearing aid fitting with MEE/AOM and SNHL is more complex and exemplifies how vital it is to implement the recommended guidelines for hearing rehabilitation, including parent counseling and multidisciplinary cooperation.

## Figures and Tables

**Table 1:** Average hearing thresholds for AC and BC in the study group and the control group.

Group	Freq.	500 Hz	1000 Hz	2000 Hz	4000 Hz	Group	Freq.	500 Hz	1000 Hz	2000 Hz	4000 Hz
	/Ear						/Ear				
Study group (Mixed HL)	AC Right (17 ears)	62.9 (13.7)	66.9 (14.4)	71.9 (13.1)	78.1 (15.5)	Control group (SNHL)	AC Right (9 ears)	52.5 (20.5)	55.6 (21.6)	62.1 (23.2)	61.3 (23.3)
	BC Right (14 ears)	41.7 (13.7)	43.4 (9.5)	51.1 (12.7)	51.2 (15.4)		BC Right (6 ears)	42.5 (19.2)	51.0 (19.5)	57.0 (12.2)	57.0 (17.2)
	AC Left (13 ears)	55.6 (19.2)	58.3 (15.5)	63.9 (14.6)	66.0 (16.8)		AC Left (7 ears)	47.9 (6.9)	52.1 (6.8)	55.7 (10.8)	59.3 (12.5)
	BC Left (7 ears)	33.6 (8.5)	40.8 (6.6)	46.4 (9.4)	43.6 (4.6)		BC Left (7 ears)	40.0 (13.2)	45.0 (12.9)	50.7 (10.3)	55.7 (14.0)

**Table 2:** Etiology of HL in both groups of children.

Etiology	Study Group, n = 18 (Children with MEE)	Control Group, n = 10 (Children without MEE)
Genetic	8	5
Cytomegalovirus	1	1
Syndrome	1	0
Birth complication	3	1
Unknown	3	3
General developmental delay	2	0

## Data Availability

No new data were created.
